# Investigation of the Transport Pathways Associated with Enhanced Brain Delivery of Peptide Drugs by Intranasal Coadministration with Penetratin

**DOI:** 10.3390/pharmaceutics13111745

**Published:** 2021-10-20

**Authors:** Noriyasu Kamei, Susumu Suwabe, Kenji Arime, Hidemi Bando, Kaho Murata, Maika Yamaguchi, Natsuki Yokoyama, Erina Tanaka, Ayaka Hashimoto, Takanori Kanazawa, Yukio Ago, Mariko Takeda-Morishita

**Affiliations:** 1 Laboratory of Drug Delivery Systems, Faculty of Pharmaceutical Sciences, Kobe Gakuin University, 1-1-3 Minatojima, Chuo-ku, Kobe, Hyogo 650-8586, Japan; pprkq048@s.kobegakuin.ac.jp (S.S.); ppyjs090@s.kobegakuin.ac.jp (K.A.); pqcfm147@s.kobegakuin.ac.jp (H.B.); prttu145@s.kobegakuin.ac.jp (K.M.); prjjo223@s.kobegakuin.ac.jp (M.Y.); profn233@s.kobegakuin.ac.jp (N.Y.); pqvpe174@s.kobegakuin.ac.jp (E.T.); ppkro044@s.kobegakuin.ac.jp (A.H.); mmtakeda@pharm.kobegakuin.ac.jp (M.T.-M.); 2 Department of Pharmaceutical Engineering and Drug Delivery Sciences, School of Pharmaceutical Sciences, University of Shizuoka, 52-1 Yada, Suruga-ku, Shizuoka 422-8526, Japan; t.kanazawa@u-shizuoka-ken.ac.jp; 3 Department of Cellular and Molecular Pharmacology, Graduate School of Biomedical and Health Sciences, Hiroshima University, Hiroshima, Hiroshima 734-8553, Japan; yukioago@hiroshima-u.ac.jp

**Keywords:** nose-to-brain delivery, exendin-4, insulin, cell-penetrating peptide, olfactory mucosa, hippocampus

## Abstract

We previously found that coadministering peptides and proteins with the cell-penetrating peptide L-penetratin intranasally significantly increased transport to the brain and enhanced pharmacological effects. The present study aimed to clarify the mechanisms of nose-to-brain drug delivery enhancement by L-penetratin coadministration. First, we compared the concentrations of Exendin-4 in plasma and brain after intranasal and subcutaneous administration and suggested that coadministration with L-penetratin facilitated the direct nose-to-brain transport of Exendin-4. Second, we demonstrated that L-penetratin did not stimulate the transport of Cy7-labeled Exendin-4 and insulin through the trigeminal nerves but shifted their distribution to the olfactory mucosal pathway. Third, we investigated the distribution of insulin into the deeper regions of the brain after delivery via the olfactory pathway and suggested that insulin had entered the olfactory bulb, bottom part of the brain, and perivascular space through the cerebrospinal fluid and had diffused throughout the brain. We further demonstrated that intranasally delivered insulin with L-penetratin specifically accumulated on the hippocampus neuronal cells. Thus, this study suggested that administrating peptide drugs intranasally with L-penetratin allows direct transport to the olfactory bulb, bottom part of the brain, and perivascular space of the cerebral artery. This technique also potentially allows targeting of specific brain areas.

## 1. Introduction

The brain is a sanctuary protected from the external environment by the blood–brain barrier (BBB), composed of microvascular endothelial cells, astrocytes, pericytes, and others, which drastically limits the influx of drugs from systemic circulation to the brain parenchyma [1]. Therefore, most therapeutic drugs cannot be delivered to the brain via peripheral administration routes such as intravenous and subcutaneous (s.c.) injections [2]. Neurotrophic proteins and nucleic acids such as mRNA and siRNA could potentially treat neurodegenerative disorders, cerebral ischemic injuries, and brain tumors. However, their bulky structures and high sensitivity to enzymatic degradation prevent them from penetrating the brain. Achieving pharmaceutical effects in the central nervous system requires the development of effective drug delivery strategies.

Intranasal administration is an attractive route for drug delivery to the brain because it allows the direct transport of drugs from the nasal cavity to the brain parenchyma by bypassing systemic circulation [3,4,5,6,7,8]. This strategy has been called “nose-to-brain delivery”. However, we and others previously clarified that intranasal administration cannot efficiently deliver macromolecular compounds such as peptides and proteins to the brain [9,10,11]. Therefore, we have developed a strategy to considerably enhance nose-to-brain delivery of macromolecular drugs by coadministering them with a cell-penetrating peptide (CPP), L-penetratin, which enhances membrane permeation [9]. Using this strategy, we successfully delivered several drugs, including insulin, glucagon-like peptide-1 (GLP-1) receptor agonist (Exendin-4), and leptin, to the brain and observed their pharmacological actions on the brain such as memory improvement and appetite reduction [9,12,13,14,15]. However, we have not clarified the mechanisms of L-penetratin-enhanced macromolecular drug brain delivery, and our previous data showed that intranasal coadministration with L-penetratin increased the systemic absorption of drugs as well as delivery to the brain [9,14,15].

Therefore, the present study evaluated the possible mechanisms related to CPP-mediated nose-to-brain delivery of macromolecules, particularly peptide drugs, through the following three steps. First, we clarified the possible contribution of direct or indirect nose-to-brain transport of peptide drugs by coadministration with L-penetratin. Second, we analyzed the dominant contribution of L-penetratin coadministration to drug transport via the olfactory mucosal or trigeminal axonal route. Third, we investigated the distribution of peptide drugs in the brain after delivery by coadministration with L-penetratin. Throughout the study, we aimed to comprehensively understand the journey of peptide drugs coadministered with L-penetratin from the nasal cavity to the targeted part of the brain.

## 2. Materials and Methods

### 2.1. Materials

Exendin-4 (HGEGTFTSDLSKQMEEEAVRLFIEWLKNGGPSSGAPPPS-NH2, 4189.7 Da) and L-penetratin (RQIKIWFQNRRMKWKK, 2247.3 Da) were synthesized by Cosmo-Bio Co., Ltd. (Tokyo, Japan). We purchased recombinant human insulin (27.5 IU/mg), 4% paraformaldehyde (PFA) in phosphate buffer, acetone, and sucrose from FUJIFILM Wako Pure Chemical Corp. (Osaka, Japan). We obtained the Exendin-4 EIA kit from Phoenix Pharmaceuticals Inc. (Burlingame, CA, USA). Cytiva (Tokyo, Japan) provided the Cy7 Mono NHS Ester. We bought a nonfluorescent rodent diet (D10001) from Research Diets Inc. (New Brunswick, NJ, USA). We purchased methyl cellulose (METOLOSE) from Shin-Etsu Chemical Co., Ltd. (Tokyo, Japan). We obtained sodium pentobarbital (Somnopentyl) from Kyoritsu Seiyaku Corp. (Tokyo, Japan). We obtained the anti-insulin rabbit monoclonal antibody (ab181547) and Alexa 488-labeled anti-rabbit IgG (H&L) goat polyclonal secondary antibody (ab150077) from Abcam plc (Cambridge, UK). We obtained Blocking One Histo from Nacalai Tesque Inc. (Kyoto, Japan). We purchased the Pierce Bicinchoninic Acid (BCA) Protein Assay Kit and ProLong Diamond Antifade Mountant with DAPI from Thermo Fisher Scientific Inc. (Waltham, MA, USA). We bought *Lycopersicon esculentum* Lectin-DyLight 594 Conjugate (Tomato Lectin) from Vector Laboratories, Inc. (Burlingame, CA, USA). We obtained triton X-100 from Merck KGaA (Darmstadt, Germany). Sakura Finetek Japan Co., Ltd. (Tokyo, Japan), provided Tissue-Tek O.C.T. Compound and Tissue-Tek Cryo-Mold #3. The low-profile disposable blades (DB80LS) came from Leica Biosystems Inc., Division of Leica Microsystems Inc. (Buffalo Grove, IL, USA). We purchased MAS-coated Slide Glass and NEO cover glass (24 × 60 mm) from Matsunami Glass Ind., Ltd. (Osaka, Japan), and Super PAP Pen (Liquid Blocker) from Daido Sangyo Co., Ltd. (Tokyo, Japan). All other chemicals were of analytical grade, and they are commercially available.

### 2.2. Animals

This study was performed at Kobe Gakuin University and complied with the regulations of the Committee on Ethics in the Care and Use of Laboratory Animals. We purchased male ddY mice and male Sprague-Dawley rats from Japan SLC Inc. (Shizuoka, Japan). We housed all rodents in rooms maintained at 23 ± 1 °C and 55 ± 5% relative humidity under a 12 h light/dark cycle and gave them free access to water and food during the acclimatization period.

### 2.3. Examination of Systemic Absorption and Brain Distribution of Peptide Drugs after Intranasal and Subcutaneous Administration

#### 2.3.1. Preparation of the Exendin-4 and L-penetratin Solutions

We dissolved Exendin-4 and L-penetratin separately in phosphate-buffered saline (PBS, pH 6.0) containing 0.001% methyl cellulose to obtain 5 mg/mL and 4.0 mM stock solutions, respectively. We then gently mixed equal volumes of the Exendin-4 and L-penetratin stock solutions and adjusted the respective concentrations to 2.5 mg/mL and 2.0 mM for intranasal administration. After mixing, the prepared Exendin-4 and L-penetratin solutions were clear. We diluted the Exendin-4 stock solution to 0.05 mg/mL with PBS (pH 7.4) containing 0.001% methyl cellulose for subcutaneous administration.

#### 2.3.2. Intranasal and Subcutaneous Administration Study

We anesthetized male ddY mice (seven weeks old) with an intraperitoneal (i.p.) injection of sodium pentobarbital (Somnopentyl, 50 mg/kg) and restrained them in a supine position on a thermostatically controlled board at 37 °C. Mice in the intranasal administration groups received 5 μL of Exendin-4 solution (2.5 mg/mL) with or without L-penetratin (2.0 mM) through a micropipette (Pipetman P-20, Gilson Inc., Middleton, WI, USA) inserted directly into their left nostril. Mice in the subcutaneous administration group received 100 μL of Exendin-4 solution (0.05 mg/mL) via subcutaneous injection. The Exendin-4 doses were 0.3125 and 0.125 mg/kg body weight for intranasal and subcutaneous administrations, respectively. At the designated time points after administration (15, 30, 60, and 90 min), we collected 0.2 mL of blood from the left jugular vein of the mice. We opened the abdominal cavity and flushed the intravascular content in the brain by perfusion of ice-cold PBS (pH 7.4) into the left ventricle of the heart at 5.5 mL/min for 5 min using a peristaltic pump (ATTO Corp., Tokyo, Japan). We quickly decapitated the mice and separated the whole brain samples into olfactory bulbs, hippocampus, cerebral cortex, and remaining portion on ice. We weighed the samples (olfactory bulbs, hippocampus, and cerebral cortex) and homogenized them with four times their volume in ice-cold assay buffer (Phoenix Pharmaceuticals, Inc., Burlingame, CA, USA) using a glass or microtube tissue grinder. We centrifuged the blood samples and homogenized samples at 4 °C and 5400× *g* for 15 min and determined the Exendin-4 concentrations in the resultant plasma or homogenate supernatant using an ELISA kit (Exendin-4 EIA kit, Phoenix Pharmaceuticals, Inc., Burlingame, CA, USA). We measured their absorbance at λmax of 450 nm using a Synergy HT microplate reader (BioTek Instruments Inc., Winooski, VT, USA).

### 2.4. Examination of Brain and Trigeminal Nerve Distribution after Intranasal Administration of Cy7-Labeled Peptide Drugs

#### 2.4.1. Labeling Exendin-4 and Insulin with the Cy7 Fluorescent Dye

We dissolved the peptides (Exendin-4 or insulin) in PBS (pH 7.4) containing 0.001% methyl cellulose. We then desalted 3 mL of Exendin-4 solution (1.67 mg/mL) or 2 mL of insulin solution (5 mg/mL) using a PD-10 column (Cytiva, Tokyo, Japan). We eluted the peptides with a 50 mM NaHCO_3_ solution and collected the eluted fractions containing the peptides. We quantified Exendin-4 by BCA assay and insulin using high-performance liquid chromatography (HPLC, on a LaChrom Elite system (Hitachi High-Tech Corp., Tokyo, Japan) with an analytical column (ChemcoPak NUCLEOSIL 5C18, 5 μm, 4.6 × 150 mm, Chemco Plus Scientific Co., Ltd., Osaka, Japan)). We obtained 3.5–6.0 mL of Exendin-4 solution at 1.16 mg/mL and 3.0–6.0 mL of insulin solution at 3.24 mg/mL. We dissolved Cy7-NHS ester (1 mg) in 0.125 mL of dimethyl sulfoxide and added it in 1.8 mL of the Exendin-4 (2.09 mg) or insulin (5.84 mg) solution. After adding 15 μL of trimethylamine to the Cy7-ester and peptide solution, we gently stirred the mixed solution overnight in the dark at room temperature. We desalted the resulting solution (1.94 mL) using a PD-10 column, eluted it with PBS (pH 7.4) with 0.001% methyl cellulose, and then adjusted its pH to 6.0. We obtained 4.0–6.0 mL of eluted solution and quantified the peptides by BCA assay or HPLC. We used the same analytical conditions for HPLC as previously described [16]. We mixed the fractions (3.0–5.0 mL) containing high concentrations of Cy7-labeled Exendin-4 (1.33 mg/mL) or Cy7-labeled insulin (1.07 mg/mL) with an equal volume of L-penetratin solution (1.0 mM) in PBS (pH 6.0) containing 0.001% methyl cellulose just before the brain distribution study.

#### 2.4.2. *Ex Vivo* Brain and Trigeminal Nerve Imaging after Intranasal Administration

We fed male Sprague-Dawley rats (seven weeks old) a nonfluorescent rodent diet (D10001) for a week before intranasal administration to avoid the intrinsic fluorescence of tissues. We anesthetized the rats with an i.p. injection of sodium pentobarbital (Somnopentyl, 50 mg/kg) and restrained them in a supine position on a thermostatically controlled board at 37 °C. We performed the surgical procedure described by Hirai et al. [17] to close the nasal cavity. Briefly, we incised the neck and cannulated the trachea and esophagus with polyethylene tubing (Hibiki No. 6) to maintain respiration and keep the solutions in the nasal cavity, respectively. We sealed the nasopalatine ducts with medical super glue (Aron Alpha A Sankyo, Daiichi-Sankyo Co., Ltd., Tokyo, Japan) to prevent the drainage of the solutions from the nasal cavity into the oral cavity. We then inserted 50 μL of Cy7-Exendin-4 (0.67 mg/mL) or Cy7-insulin (0.5 mg/mL) solution with or without L-penetratin (0.5 mM) through a micropipette (Pipetman P-100) directly into the rats’ left nostril. At the designated time points after administration (15, 30, and 60 or 120 min), we quickly exsanguinated the rats from the postcaval vein and flushed the administered solution by perfusion of PBS (pH 7.4). We decapitated the rats, carefully isolated the whole brain and right and left trigeminal nerves, and washed them with ice-cold PBS (pH 7.4). We placed the brain samples onto a brain matrix (MK-RC-01, Muromachi Kikai Co., Ltd., Tokyo, Japan) and sliced with blades (TCB-100) into twelve 2 mm-thick fractions. We placed the sliced brain and trigeminal nerve samples on a silicon board and mounted them on the stage of an *in vivo* imaging system (IVIS Lumina XR, Caliper Life sciences, Inc., Waltham, MA, USA). We analyzed the distribution characteristics of fluorescence derived from Cy7 in the brain and trigeminal nerves at excitation and emission wavelengths of 747 and 776 nm, respectively. We quantified the fluorescence intensity of the region of interest of the images based on their contrast. To calculate the fluorescence intensity derived from Cy7 in the tissues, we subtracted the intrinsic fluorescence of an untreated area from the total intensity.

### 2.5. Histoimmunological Staining of Brain Specimens after Intranasal Administration of Peptide Drugs

We anesthetized male ddY mice (seven weeks old) with an i.p. injection of sodium pentobarbital (Somnopentyl, 50 mg/kg) and restrained them in a supine position on a thermostatically controlled board at 37 °C. We then administered them 10 μL of insulin solution (30 IU/mL) with or without L-penetratin (2.0 mM) through a micropipette (Pipetman P-20, Gilson Inc., Middleton, WI, USA) inserted directly into their left nostril. The dose of insulin was 10 IU/kg body weight (30 IU/mL). Thirty minutes after intranasal administration, we opened the abdominal cavity and flushed the intravascular content in the brain by perfusion of ice-cold PBS (pH 7.4) into the left ventricle of the heart at 5.5 mL/min for 5 min using a peristaltic pump (ATTO Corp.). We immediately fixed the brain by perfusion of ice-cold 4% PFA into the brain at 5.5 mL/min for 10 min. We next carefully isolated the whole brain and immersed it in 4% PFA for 24 h and then in PBS (pH 7.4) containing 20% sucrose at 4 °C for 24 h twice. After removing the sucrose-PBS, we placed the brain specimen on Cryo-Mold #3 and rapidly embedded it in O.C.T. compound on dry ice with acetone. We prepared thin slices with 50 μm thickness at bregma 4.25, 0.85, and −1.91 mm from the frozen brain using a cryostat (CM3050S, Leica Microsystems Inc, Buffalo Grove, IL, USA). We mounted them on a glass slide and washed them with 40 μL of PBS (pH 7.4) containing 0.3% Triton X-100 (t-PBS) three times for 5 min. We treated the slices with a drop of blocking reagent (Blocking One Histo) for 10 min at room temperature to inhibit nonspecific reactions. We incubated the slices with 40 μL of anti-insulin antibody solution (1:1000, 1 μg/mL) overnight at 4 °C. After washing them with t-PBS three times, we treated the slices with 40 μL of Alexa 488-labeled goat anti-rabbit IgG solution (1:1000, 2 μg/mL) for 1 h at room temperature. We then washed the slices with t-PBS three times and incubated them with 40 μL of DyLight 594-tomato lectin solution (1:200, 5 μg/mL) for 30 min at room temperature. Finally, we washed them with t-PBS three times and treated them with 20 μL of mounting medium containing antifade reagent and DAPI (ProLong Diamond Antifade Mountant with DAPI, Thermo Fisher Scientific Inc.). We observed the stained brain tissue by confocal laser scanning microscopy (FV3000, Olympus Corp., Tokyo, Japan).

### 2.6. Statistical Analysis

We expressed data as the mean and standard error of the mean (SEM) of multiple determinations. We evaluated the significance of the differences in the mean values of two groups using Student’s unpaired *t*-test. We performed statistical analyses with IBM SPSS Statistics Version 23 (IBM Corp., Armonk, NY, USA). We considered differences as significant when the *p* value < 0.05.

## 3. Results

### 3.1. Contribution of Direct Nose-to-Brain Transport to the L-Penetratin-Enhanced Brain Delivery of Exendin-4

We previously demonstrated that intranasal coadministration with L-penetratin, a typical amphipathic CPP, significantly increased the delivery of insulin, Exendin-4, and leptin to the brain [9,12,14,15]. During the permeation through the nasal mucosa, drugs come across microvascular vessels in the lamina propria directed toward the systemic circulation before reaching the brain or cerebrospinal fluid (CSF) [3]. Our previous data showed that intranasal coadministration with L-penetratin elevated the drugs’ plasma concentration more than their brain concentration [9,14,15], suggesting that the predominant factor for improved brain distribution is the higher absorption into the systemic circulation rather than direct nose-to-brain transport. Therefore, we first examined the contribution of direct nose-to-brain delivery and indirect distribution via systemic circulation to the delivery efficiency of coadministration with L-penetratin.

We used Exendin-4 as a model peptide because we previously showed that its coadministration with L-penetratin resulted in efficient brain delivery and blood absorption [14]. We administered Exendin-4 intranasally (with or without L-penetratin) and subcutaneously to mice and compared the Exendin-4 concentrations in the plasma and brain.

**Figure 1:**
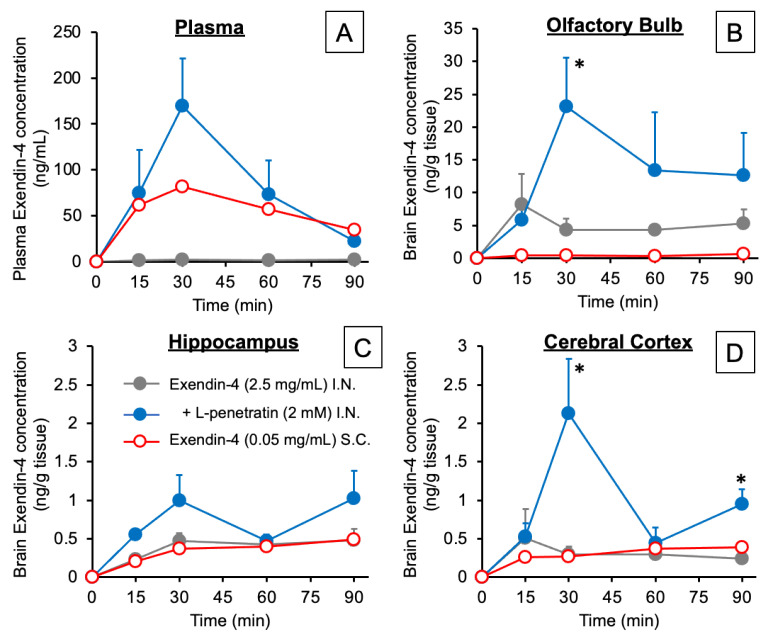
Time-course profiles of Exendin-4 concentrations in plasma (**A**), olfactory bulbs (**B**), hippocampus (**C**), and cerebral cortex (**D**) after intranasal or subcutaneous administration to mice. The intranasal Exendin-4 administration dose was 0.3125 mg/kg (2.5 mg/mL, 5 μL/40 g mouse) with or without L-penetratin (2 mM), and the subcutaneous injection dose was 0.125 mg/kg (0.05 mg/mL, 100 μL/40 g mouse) without L-penetratin. Data are expressed as the mean ± SEM of *n* = 3–5. * indicates a significant difference (*p* < 0.05) from the control intranasal administration group without L-penetratin.

**Figure 2:**
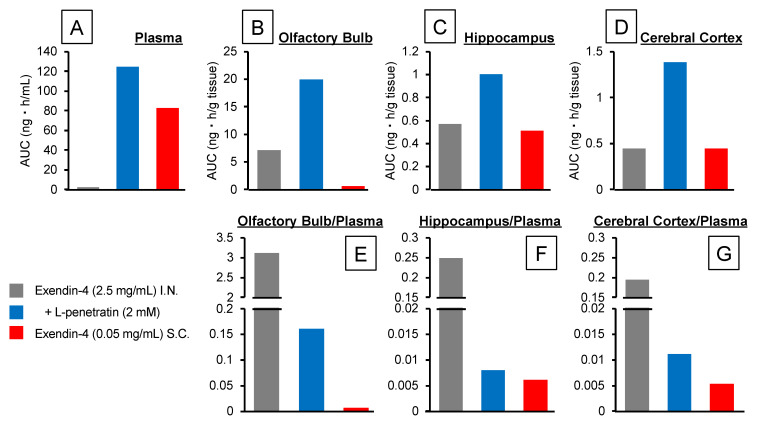
Area under the concentration-time curve (AUC) calculated with the time-course profiles of Exendin-4 concentration after intranasal or subcutaneous administration to mice. Panels (**A**–**D**) show the AUCs in plasma, olfactory bulbs, hippocampus, and cerebral cortex, respectively. Panels (**E**–**G**) show the ratios of AUCs derived from the olfactory bulbs, hippocampus, and cerebral cortex per plasma, respectively.

Figure 1A and Figure 2A, respectively, show the time-course profiles of Exendin-4 plasma concentrations and the calculated AUCs after intranasal or s.c. administration. We detected no plasma Exendin-4 after intranasal administration without L-penetratin, but a significant amount was absorbed into the circulation when coadministered with L-penetratin. We adjusted the Exendin-4 dose for s.c. injection to get plasma levels similar to those obtained after intranasal administration with L-penetratin. As shown in Figure 1B–D and Figure 2B–D, we detected high Exendin-4 levels in brain regions such as the olfactory bulb, hippocampus, and cerebral cortex after intranasal administration with L-penetratin and low levels in the olfactory bulb even without L-penetratin (Figure 1B and Figure 2B). By contrast, the brain concentration of Exendin-4 after s.c. injection was much lower than that after intranasal administration (Figure 1B,D and Figure 2B,D). The high plasma Exendin-4 level was not related to the increase of the brain level, suggesting that direct nose-to-brain delivery mediated the significant increase of Exendin-4 concentration in the brain after intranasal administration with L-penetratin. Conversely, Figure 2E,F show that coadministration of Exendin-4 with L-penetratin decreased the brain/plasma AUC ratio because L-penetratin enhanced the systemic absorption of Exendin-4 more strongly than its transport to the brain. However, the brain/plasma AUC ratio of Exendin-4 after intranasal administration with L-penetratin was higher than that after s.c. administration, and L-penetratin significantly increased the absolute amount delivered to the brain. Thus, intranasal coadministration with L-penetratin is useful in delivering peptide drugs directly to the brain.

### 3.2. Comparison of the Contribution of Olfactory Mucosal Transport and Trigeminal Axonal Transport on L-Penetratin-Enhanced Peptide Brain Delivery

The results above suggest that L-penetratin enhances the delivery of peptide drugs via a direct nose-to-brain transport pathway. Earlier publications proposed that nose-to-brain drug transport could involve several routes, such as olfactory mucosal routes along with the olfactory sensory neurons to the olfactory bulb and trigeminal axonal transport to the brainstem [3,5,18,19]. Therefore, we next investigated the more detailed mechanisms associated with the effect of L-penetratin on the nose-to-brain drug transport by analyzing the distribution of peptide drugs in the brain and trigeminal nerves.

**Figure 3:**
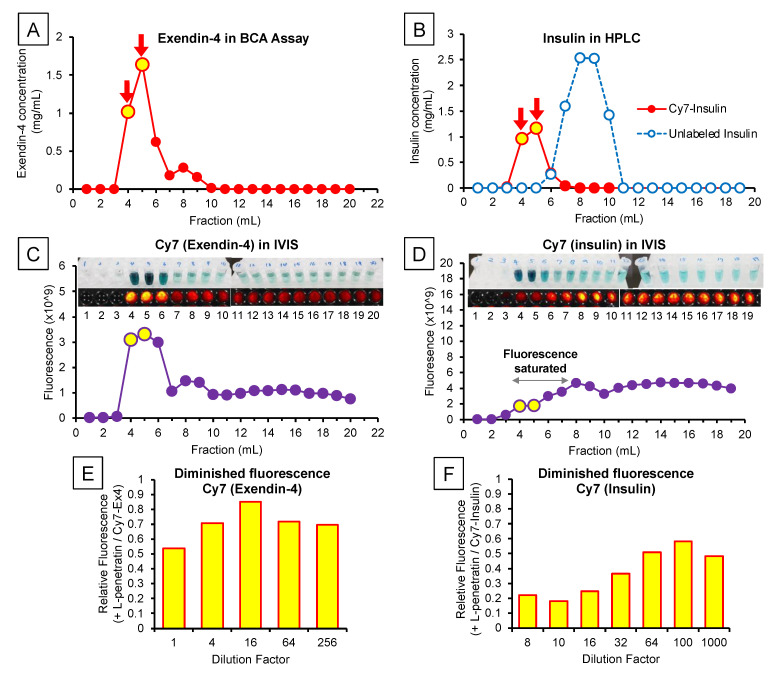
Characterization of the peptides labeled with a fluorescent probe (Cy7). Panels (**A**) and (**B**), respectively, show the concentrations of Exendin-4 (BCA assay) and insulin (HPLC) in the eluted fractions after labeling with Cy7. Panels (**C**,**D**) show the fluorescence intensity in the eluted fractions measured by the *in vivo* imaging system and the reference photos of these samples. The fluorescence intensity of fractions 4–7 of Cy7-labeled insulin exceeded the upper limit of detection, in contrast with the visible fluorescence shown in the photos (panel (**D**)). Panels (**E**,**F**) indicate the weakened fluorescence of Cy7-labeled Exendin-4 and insulin mixed with L-penetratin. L-penetratin reduced the fluorescence of Cy7-labeled Exendin-4 and insulin to 54% and 22% of that without L-penetratin, respectively. Dilution allowed partial fluorescence recovery to 70% and 48%.

In this examination, we measured the fluorescently labeled Exendin-4 and insulin in rat brain slices and trigeminal nerves using an *in vivo* imaging system under *ex vivo* conditions. As Figure 3A–D show, we labeled Exendin-4 and insulin with Cy7 and purified them. However, we confirmed that mixing Cy7-Exendin-4 or insulin with L-penetratin complicates the accurate quantification of Cy7 fluorescence as it decreased the fluorescence intensity of Cy7-Exendin-4 and Cy7-insulin to 54% and 22%, respectively (Figure 3E,F) [20]. We confirmed that diluting the complex with PBS allowed the recovery of 70% (Cy7-Exendin-4) and 48% (Cy7-insulin) of the fluorescence intensity. Therefore, we corrected the quantitative data obtained with the *in vivo* imaging system after coadministration of Cy7-Exendin-4 and Cy7-insulin with L-penetratin based on the reduction ratio (70% and 48%, respectively).

**Figure 4:**
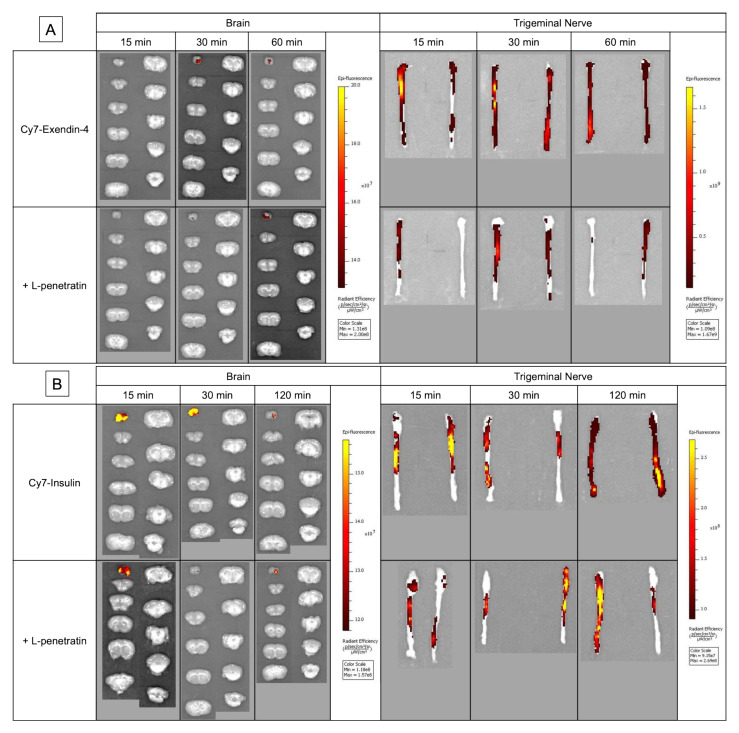
*Ex vivo* fluorescence imaging of brain slices and trigeminal nerve bundles after intranasal administration of Cy7-labeled Exendin-4 (panel (**A**)) and insulin (panel (**B**)) with or without L-penetratin to Sprague-Dawley rats. The tissues were isolated at 15, 30, and 60 or 120 min after administration, and fluorescence was measured with the same settings for all treatment groups. The images show a typical result obtained in one experiment. Separate experiments under the same conditions yielded similar results (*n* = 3–5).

**Figure 5:**
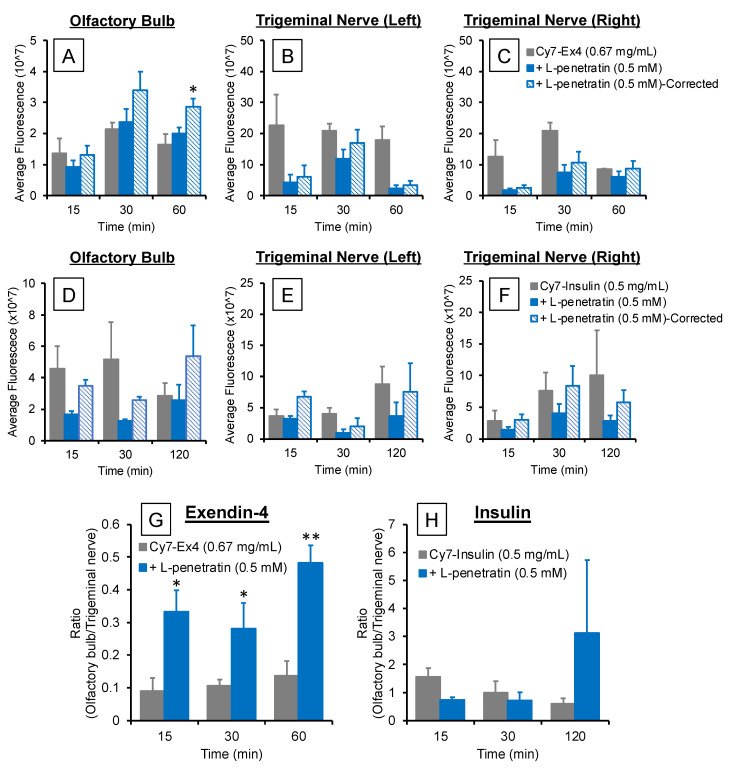
Fluorescence intensity in the olfactory bulbs (panels (**A**,**D**)) and left and right trigeminal nerves (panels (**B**,**C**,**E**,**F**)) after intranasal administration of Cy7-labeled Exendin-4 and insulin with or without L-penetratin calculated from the imaging data presented in Figure 4. We corrected the L-penetratin-induced Cy7 fluorescence loss using the relative fluorescence shown in Figure 3E,F (70% for Cy7-Exendin-4 and 48% for Cy7-insulin). Panels G and H show the fluorescence ratios of olfactory bulbs per trigeminal nerves at 15, 30, and 60 or 120 min after intranasal administration of Cy7-Exendin-4 (**G**) and Cy7-insulin (**H**) with or without L-penetratin. Data are expressed as the mean ± SEM of *n* = 3–5. * (*p* < 0.05) and ** (*p* < 0.01) indicate a significant difference from the control intranasal administration group without L-penetratin.

Figure 4A,B show the *ex vivo* images of the rat brain slices and trigeminal nerve bundles isolated after intranasal administration of Cy7-Exendin-4 and Cy7-insulin with or without L-penetratin. We detected fluorescence signals in the olfactory bulb and trigeminal nerves, consistent with the mechanisms associated with the olfactory mucosal route and trigeminal axonal route proposed in previous reports [3,5,12,19]. Figure 5A–F show quantitative data derived from the olfactory bulb and trigeminal nerve images (Figure 4). As expected, because L-penetratin reduced the fluorescent signals of Cy7-Exendin and Cy7-insulin, coadministration of L-penetratin with the Cy7-labeled peptides reduced the fluorescence intensity in the olfactory bulb and trigeminal nerves. To address this issue, we corrected the fluorescence values after coadministration with L-penetratin using the diminished fluorescence ratio calculated in Figure 3E,F. However, the corrected fluorescence values did not demonstrate an increase in Cy7-Exendin-4 and Cy7-insulin fluorescence in the trigeminal nerves (Figure 5B,C,E,F). By contrast, coadministration with L-penetratin gradually elevated the fluorescence in the olfactory bulb (Figure 5A,D). Figure 5G,H show the distribution ratio (olfactory bulb/trigeminal nerves) after intranasal administration of Cy7-Exendin-4 and Cy7-insulin with or without L-penetratin. Thus, coadministration with L-penetratin dominantly increased the peptides’ distribution to the olfactory bulb rather than to the trigeminal nerves. Because the distribution of peptides to the trigeminal nerves did not contribute to the delivery to the posterior parts of the brain such as the brainstem (Figure 4), coadministration with L-penetratin might enhance the brain delivery of peptide drugs by shifting their trigeminal axonal distribution to the transport to the olfactory mucosa and olfactory bulb.

### 3.3. Detailed Evaluation of the Distribution of Peptide Drugs Delivered to the Brain by Coadministration with L-Penetratin

The results above suggested that L-penetratin can directly deliver the peptide drugs from the nasal cavity to the brain parenchyma through olfactory mucosal transport. However, even though drugs can effectively be delivered from the nasal cavity to the olfactory bulb, which is an anterior part of the brain, further diffusion throughout the brain is probably difficult. Recent publications suggested that bulk flow from the lamina propria to the CSF through the perineuronal and perivascular pathways can achieve olfactory transport of drugs to the brain [3,21,22]. We previously suggested that insulin, administered intranasally with L-penetratin, can reach the CSF, but the concentration was higher around the anterior part of the brain than around its posterior part [12]. Recent findings suggested that the glymphatic (glial-lymphatic) pathway plays a critical role in drug delivery to the CSF or interstitial fluid in the brain through the perivascular space [21,22,23,24,25]. Therefore, we next evaluated the peptides’ distribution from the olfactory bulb or CSF into the deeper part of the brain.

**Figure 6:**
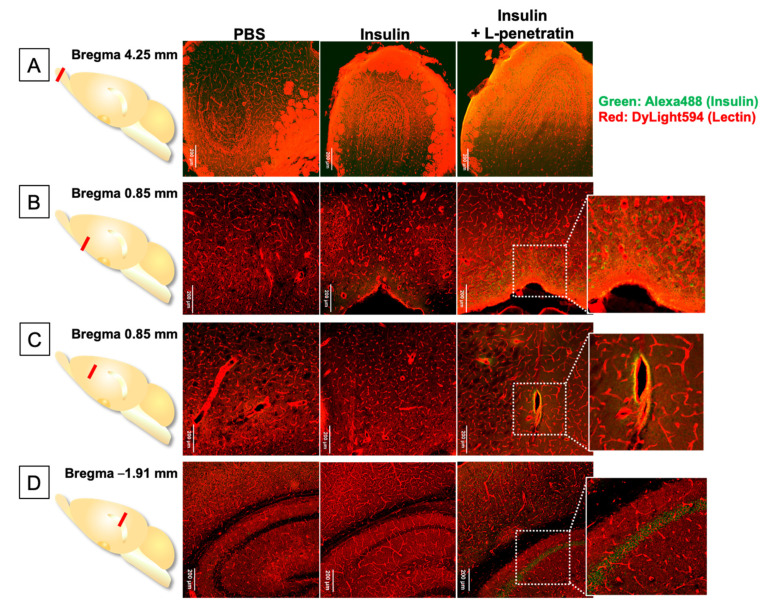
Immunohistological staining of brain sections after intranasal administration of insulin with or without L-penetratin to mice. The brains were isolated 30 min after intranasal administration of PBS (negative control) or insulin in the absence or presence of L-penetratin. (**A**) Olfactory bulbs, (**B**) bottom part of the brain, (**C**) central part, and (**D**) cerebral cortex and hippocampus (CA2) at 4.25, 0.85, 0.85, and −1.91 mm from bregma. Insulin was detected with an anti-insulin primary antibody and Alexa 488-conjugated secondary antibody (green). Blood vessels were stained with DyLight 594 conjugated tomato lectin (red). The images show a typical result obtained in one experiment. Separate experiments under the same conditions yielded similar results (*n* = 3 or 4).

In this examination, we used insulin as a model peptide drug and administered it intranasally with L-penetratin to mice. Figure 6A–D show the immunohistological staining of the brain slices at 4.25 mm (olfactory bulb), 0.85 mm (around the hypothalamus and upper part), and −1.91 mm (hippocampus) from bregma. As shown in Figure 6A, most of the bright fluorescence derived from insulin came from the surface of the olfactory bulb after coadministration of insulin with L-penetratin. Moreover, Figure 6B shows insulin-derived intensive fluorescence at the bottom part of the brain (bregma 0.85 mm), consistent with our previous autoradiography results [12]. As shown in Figure 6C, after coadministration with L-penetratin, further observation at bregma 0.85 mm revealed the presence of insulin at the perivascular spaces of arterial blood vessels. Thus, insulin administered intranasally with L-penetratin could first enter the CSF around the olfactory bulb, could be distributed mainly into the olfactory bulb, and could move gradually along with CSF and could be distributed to the bottom part of the brain. Furthermore, insulin at the lamina propria or CSF could enter the perivascular space and follow the artery to the deeper part of the brain. As shown in Figure 6D, further examination around the hippocampus at bregma −1.91 mm clarified that insulin could reach the deeper part of the brain, around the hippocampus, after coadministration with L-penetratin.

**Figure 7:**
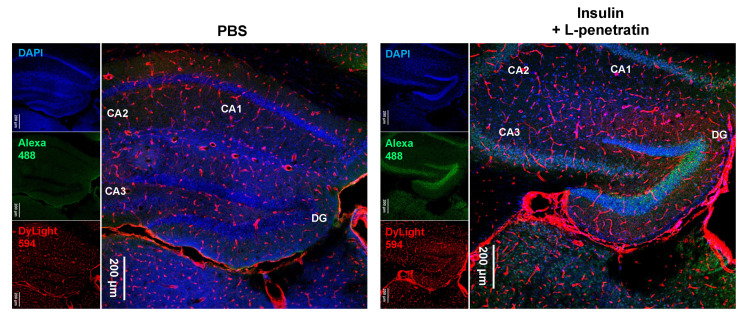
Immunohistological staining of the hippocampus at −1.91 mm from bregma after intranasal administration of PBS or insulin with L-penetratin to mice. Insulin was detected with an anti-insulin primary antibody and Alexa 488-conjugated secondary antibody (green). Blood vessels were stained with DyLight 594 conjugated tomato lectin (red). The images show a typical result obtained in one experiment. Separate experiments under the same conditions yielded similar results (*n* = 3 or 4).

We next investigated the detailed insulin localization. Figure 7 shows that insulin delivered into the brain by the effect of L-penetratin can specifically accumulate on the pyramidal cells of the hippocampus and the granular cells of the dentate gyrus. This hippocampus-specific insulin accumulation could explain the improvement of memory by intranasal coadministration of insulin with L-penetratin in senescence-accelerated mice [13].

## 4. Discussion

Intranasal administration is currently an attractive strategy for drug delivery to the brain [3,4,8,26,27,28]. This approach enables the direct transport of drugs from the nasal cavity to the brain parenchyma by bypassing the BBB, which strictly limits drug influx to the brain. Therefore, it does not require drug delivery systems facilitating permeation through the BBB and avoids systemic side effects. Many reports have suggested that intranasal administration is applicable to various drugs, including peptides and proteins. However, our previous data established that the delivery of macromolecular drugs to the brain was less efficient via intranasal administration than via intravenous injection [9]. Therefore, we have proposed the coadministration with CPPs for efficient nose-to-brain delivery and demonstrated that the amphipathic CPP L-penetratin significantly increased the brain delivery of peptides and proteins such as insulin, Exendin-4, and leptin via intranasal administration [9,14,15]. Furthermore, the drugs delivered to the brain by the effect of L-penetratin exerted their pharmacological activities in the brain, such as memory and learning improvement and appetite lowering [13,14,15].

**Figure 8:**
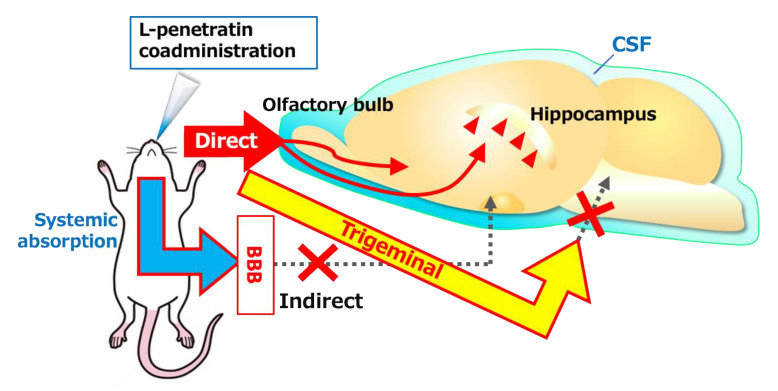
Schematic of the mechanisms associated with the transport of peptide drugs to the brain after intranasal coadministration with CPPs. The peptides travel directly from the nasal cavity to the olfactory bulb or CSF and further diffuse through the olfactory bulb or CSF or are distributed via perivascular spaces deeper into the brain. The fractions of the peptide drugs absorbed systemically or transported to the trigeminal nerves cannot contribute to the distribution to the brain. Insulin delivered to the brain by coadministration with CPPs can accumulate in the hippocampus.

Besides the advantages of nose-to-brain delivery, we found that intranasal coadministration with L-penetratin also significantly increased the systemic absorption of drugs [9,14,15]. Therefore, we had to confirm that L-penetratin could facilitate the direct nose-to-brain transport of drugs and that the systemically absorbed drugs did not simply cross the BBB. In the first investigation, we demonstrated that increasing the plasma concentration of Exendin-4 did not elevate the brain concentration by evaluating the pharmacokinetics after s.c. injection (Figure 1 and Figure 2). This suggested that the coadministration with L-penetratin enhanced the direct nose-to-brain delivery of Exendin-4 (Figure 8). Ideally, selectively delivering the drugs to the brain and avoiding systemic absorption would prevent potential systemic side effects.

Although we clarified the direct nose-to-brain delivery of peptide drugs by coadministration with L-penetratin, the mechanisms of L-penetratin-enhanced nose-to-brain transport have remained unclear. Many studies suggested that drugs could travel via the olfactory sensory neurons from the olfactory mucosa to the olfactory bulb, but this is unlikely because this movement requires long transport times (over 24 h) [3,29,30]. Conversely, drugs can cross the space around olfactory nerve bundles, can enter the CSF and the perivascular spaces in the lamina propria, and can be delivered into the brain [21,22,25]. Furthermore, several reports suggested that trigeminal nerves contribute to the transport from the nasal mucosa to the posterior parts of the brain, such as the brainstem [5,18,19]. Therefore, we next investigated the transport route of drugs coadministered with L-penetratin by detecting the fluorescence-labeled peptides (Cy7-Exendin-4 and Cy7-insulin) in brain slices and trigeminal nerves. As shown in Figure 5A,D, the distribution of Cy7-Exendin-4 and Cy-insulin in the olfactory bulb increased 60 and 120 min after administration with L-penetratin. By contrast, the distribution of Cy7-Exendin-4 and Cy7-insulin in the trigeminal nerves was high even without L-penetratin (Figure 5B, C, E, and F). Therefore, the effect of L-penetratin on the trigeminal nerve distribution of drugs was difficult to assess. Additionally, Figure 4A,B show that the intrinsically higher distribution of peptide drugs in the trigeminal nerves after intranasal administration did not lead to further distribution to the anterior part of the brain. These *ex vivo* examinations suggested that the trigeminal axonal transport pathway did not contribute to the nose-to-brain delivery of peptides and proteins. Moreover, coadministration with L-penetratin shifted the trigeminal distribution of peptide drugs to the olfactory mucosal transport to the olfactory bulb or CSF (Figure 8).

Thus, we determined that intranasal coadministration of peptide drugs and L-penetratin achieves direct nose-to-brain delivery through the olfactory mucosa. However, the peptides’ distribution from the olfactory bulb or CSF to the deeper part of the brain remained unclear. We therefore examined it more accurately by conducting immunohistological staining experiments. We used insulin as a model peptide drug and coadministered it with L-penetratin intranasally to mice. We then evaluated insulin distribution in brain slices at 4.25, 0.85, and −1.91 mm from bregma. The results suggested that insulin coadministered with L-penetratin diffused to the surface of the olfactory bulb and lower part of the brain around the hypothalamus, probably from the CSF (Figure 6A,B and Figure 8). This finding is consistent with our earlier autoradiographic and ELISA analyses [12], where we detected relatively high insulin levels at the bottom part of the brain and in the CSF surrounding the anterior part of the brain. The distribution of insulin to the bottom part of the brain might be associated with the high expression of insulin receptor in the hypothalamus [31,32]. Furthermore, we found an intense insulin signal at the perivascular spaces surrounding the arterial blood vessels (Figure 6C), suggesting the involvement of the glymphatic flow in peptide transport deeper into the brain. That is to say, coadministration with L-penetratin allows peptide drugs to permeate through the nasal epithelial membranes. Peptides then cross the lamina propria and cribriform plate and reach the CSF. Through this pathway, peptide drugs can enter the perivascular spaces and eventually travel to the interstitial fluid in the brain [21,22].

Moreover, the insulin that reaches the brain owing to L-penetratin coadministration accumulated around the hippocampus, specifically on the pyramidal cells in the hippocampus and the granular cells in the dentate gyrus (Figure 7 and Figure 8). These neuronal cells express the insulin receptor, which could explain the distribution of insulin in the brain. Besides the therapeutic potential of insulin against neurodegenerative disorders, insulin could be used as a carrier peptide to deliver other drugs to the hippocampus.

As summarized in Figure 8, this study clarified the transport route of peptide drugs to the brain when coadministered with L-penetratin intranasally. Coadministration of peptides with L-penetratin allows direct delivery from the nasal cavity to the olfactory bulb or CSF through the olfactory mucosal pathway. The peptides delivered to the brain can travel from the olfactory bulb surface or the bottom part of the brain around the hypothalamus to the deeper region of the brain via the CSF or perivascular spaces. Furthermore, insulin can specifically accumulate into the hippocampus, which is involved in neurodegenerative disorders such as Alzheimer’s disease. Our future work will aim to enhance drug delivery selectivity toward the brain by decreasing systemic absorption and to utilize insulin as a drug vector to target the hippocampus.

## Data Availability

Not applicable.
